# Inhibition of Focal Adhesion Restricts Chemoresistance in Pancreatic Cancer by Targeting SLC7A11 Mediated Ferroptosis

**DOI:** 10.1002/advs.75216

**Published:** 2026-04-20

**Authors:** Yingjin Wang, Hui Yang, Baoyuan Zhang, Yongsu Ma, Kuangen Zhang, Peng Wang, Jiapeng Yang, Zaiqi Wang, Rong Liu, Xiaodong Tian, Ning Zhang, Yinmo Yang

**Affiliations:** ^1^ Department of Hepatobiliary Pancreatic Surgery Peking University First Hospital Beijing China; ^2^ Translational Cancer Research Center Peking University First Hospital Beijing China; ^3^ Department of Thoracic Surgery Linyi People's Hospital Linyi Shandong China; ^4^ State Key Laboratory of Natural and Biomimetic Drugs Peking University Beijing China; ^5^ InxMed (Shanghai) Co., Ltd. Shanghai China; ^6^ International Cancer Institute Peking University Health Science Center Beijing China; ^7^ Yunnan Baiyao Group Kunming China

**Keywords:** chemoresistance, ferroptosis, focal adhesion kinase, pancreatic cancer, scRNA‐seq

## Abstract

Chemoresistance in pancreatic ductal adenocarcinoma (PDAC) is common and complex, accompanied with chemotherapy process. Gemcitabine‐based chemotherapy regimens have shown limited antitumor effects for PDAC, and combination targets are urgently needed to restrict chemoresistance. We attempted to identify the candidate targets with gemcitabine (Gem) through scRNA‐seq and bulk‐seq analysis based on chemotherapy‐treated and Gem‐resistant (GR) samples, respectively. The mechanisms were investigated with experimental validation in vitro and in vivo preclinical PDAC models. We found that chemoresistance and evolution after chemotherapy of PDAC were associated with activation of focal adhesion signal. Mechanistically, the focal adhesion kinase (FAK) inhibitor (IN10018) could restrict chemoresistance to Gem of PDAC by targeting SLC7A11‐mediated ferroptosis through PI3K‐Akt signaling pathway. For tumor microenvironment, IN10018 reduced the abundance of mesenchymal components and enhanced CD8^+^ T cell infiltration.

## Graphical abstract

FAK signaling drives chemoresistance and tumor evolution in pancreatic ductal adenocarcinoma. The FAK inhibitor IN10018 restores gemcitabine sensitivity by inducing SLC7A11‐mediated ferroptosis through the PI3K‐Akt pathway and remodeling the tumor microenvironment, reducing mesenchymal components while enhancing CD8^+^ T‐cell infiltration in preclinical models.

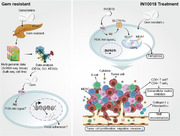

## 1 Background

Pancreatic ductal adenocarcinoma (PDAC) is expected to become the third leading cause of cancer‐related deaths by 2030, with mortality rates rising by 0.3% annually since 2000 [1]. Recently, the 5‐year survival rate has shown slight improvement, reaching 11% [2]. Chemotherapy remains the primary systemic treatment for PDAC, used in neoadjuvant, adjuvant, and palliative care settings [3]. Gemcitabine remains the standard first‐line chemotherapeutic agent for PDAC [4, 5].

Despite this, the median overall survival for patients treated with the Gemcitabine/nab‐Paclitaxel combination is only 8.6 months, while those receiving Gemcitabine alone survive for just 6.7 months [6, 7]. The objective response rates are similarly low, with Gemcitabine monotherapy achieving 8–11%, and the Gemcitabine/nab‐Paclitaxel regimen showing a response rate of around 23%. This means that approximately 70–90% of patients do not experience a significant response to palliative chemotherapy [7]. Given these poor outcomes, further exploration of the genetic and molecular mechanisms driving PDAC progression and chemoresistance is essential to identify new therapeutic targets and develop more effective treatments [7].

Mechanisms of chemoresistance in cancer can be classified as endogenous or exogenous. Endogenous resistance results from tumor cell variations and evolutionary adaptations, while exogenous resistance arises from the heterogeneity of the tumor microenvironment (TME), which includes stromal cells and the extracellular matrix (ECM). In the case of endogenous resistance, cancer cells undergo adaptive genetic and epigenetic changes under drug selection pressure, leading to the development of a permanently resistant phenotype [8]. Exogenous resistance, on the other hand, is driven by the remodeling of the TME—comprising stromal cells, immune cells, and the ECM—which promotes tumor growth, suppresses immune responses, and forms a barrier to drug delivery [9, 10]. Focal adhesion kinase (FAK), a non‐receptor cytoplasmic protein tyrosine kinase, plays a central role in regulating ECM interactions through integrins and growth factor receptors [11]. FAK inhibition (FAKi) has recently emerged as a promising strategy to reduce platinum‐based chemoresistance in ovarian cancer [12] and to enhance immunotherapy efficacy in PDAC [13]. Based on these findings, we hypothesize that FAKi may also help overcome chemoresistance in PDAC.

In this study, we tested this hypothesis using the FAK inhibitor IN10018 and uncovered a novel mechanism by which FAK inhibition impacts both endogenous and exogenous aspects of PDAC chemoresistance.

## 2 Materials and Methods

### 2.1 Cell Culture and Transfection, Drug and Assay Reagents

We cultured human PDAC cell lines, including Mia PaCa‐2, PANC‐1, Panc02, KPC, PSC, and 293T, in the central laboratory of Peking University First Hospital. All cell lines were tested for mycoplasma contamination. The cultures were maintained at 37°C with 5% CO_2_ in DMEM supplemented with 10% fetal bovine serum (FBS). Gemcitabine‐resistant (GR) Mia PaCa‐2 and PANC‐1 cells were cultured in complete medium containing 0.2 µm gemcitabine (Gemzar, Eli Lilly). Lentiviral vectors for SLC7A11 shRNA were constructed using the pLKO.1 plasmid. Transfections were performed following the manufacturer's protocol for Vigofect (Vigorous Biotechnology, Beijing, China). Sequences used for cloning are provided in Table S1. IN10018 was supplied by InxMed, while Gemcitabine, Erastin, and Fer‐1 were purchased from MedChemExpress (MCE).

### 2.2 Gemcitabine‐Resistant Cell Lines

Gemcitabine‐resistant pancreatic cancer cell lines were established using a stepwise concentration gradient method. Parental MIA PaCa‐2‐WT and PANC‐1‐WT cells were initially treated with 10 nm gemcitabine, followed by gradual dose escalation to 20, 40, 60, 150, 200, and finally 250 nm, with prolonged culture and selection at each concentration. Once the cells achieved stable growth under the corresponding gemcitabine concentration, the resistant sublines, designated as MIA PaCa‐2‐GR and PANC‐1‐GR, were obtained and subsequently preserved in liquid nitrogen.

### 2.3 Dose‑Dependent Assay and Drug Synergy Analysis

We seeded cells in 96‐well plates at an optimized density of 3 000 cells per well in 100 µl of medium containing 10% FBS. After 24 h to allow adherence, we treated the cells with the appropriate doses. Following a 72‐h exposure to gemcitabine, alone or in combination with IN10018, we processed each well with a mixture of 100 µl DMEM and CCK‐8 solution (Dojindo, Japan) at a 90:10 ratio (90 µl DMEM + 10 µl CCK‐8) at 37°C for 1 h. We then measured absorbance at 450 nm using a 96‐well plate reader and calculated relative cell numbers as percentage viability. We evaluated ten serially diluted doses of gemcitabine, testing each dose in technical triplicates per biological replicate, in a dose‐dependent assay. We calculated IC_50_ values, which correspond to 50% viability, using Prism version 7.03 (Prism Software Corporation, CA, USA). The experiment included five doses of IN10018 combined with five doses of gemcitabine, each tested in triplicates. We calculated and visualized synergy scores using SynergyFinder (RRID: SCR_019318) with the default model.

### 2.4 Colony Formation Assay

We seeded 1 000 cells into 6‐well plates, allowed them to adhere overnight, and then treated them with DMSO, IN10018 (5 µm), gemcitabine (0.5 and 0.1 µm for wild‐type [WT] cells; 0.1 and 0.15 µm for gemcitabine‐resistant [GR] cells), or combinations of these treatments for 14 days. The culture medium was refreshed every three days. After the 14‐day incubation, we washed the cells with 1× PBS and fixed them with 4% paraformaldehyde (Sigma‐Aldrich) for 20 min at room temperature. After two additional PBS washes, we stained the cells with crystal violet (0.5 g in 80 mL water and 20 mL methanol; Sigma) for 30 min at room temperature [14]. Excess stain was removed by gently washing the cells with distilled water. Each experiment was performed in triplicate.

### 2.5 Transwell Migration and Invasion Assay

For migration and invasion assays, we cultured pancreatic cancer (PC) cells overnight in serum‐free medium. We then seeded 2 × 10^4^ cells in serum‐free DMEM into the upper chambers of 24‐well Transwell inserts (Corning, NY, USA). The inserts for the invasion assay were precoated with a 1:7 dilution of Matrigel Matrix (Corning), following the manufacturer's instructions. The lower chambers contained 500 µl of DMEM with 10% FBS as a chemoattractant. The cells were cultured for 48 h under standard conditions. Afterward, we fixed the migrated cells with 4% formaldehyde, stained them with crystal violet, and counted them in five randomly selected fields at 200× magnification using a microscope. We conducted three independent experiments for quantification.

### 2.6 Wound Healing Assay

We seeded PC cells in 6‐well plates and cultured them until they reached confluence. Using a sterile 10 µl pipette tip, we introduced a wound into the cell monolayer. The cells were gently washed three times with PBS to remove detached cells and debris, then incubated in fresh serum‐free DMEM under standard conditions. At two time points (0 and 24 h), we photographed five random fields per wound at 100× magnification using a microscope. Wound width was measured with ImageJ software. Each experiment was performed in triplicate.

### 2.7 Flow Cytometry for Cell Cycle, Apoptosis and ROS Analysis

For cell cycle analysis, we treated cells with DMSO, IN10018, gemcitabine, or a combination of these agents. After 48 h, we harvested the cells using trypsin without EDTA to create a single‐cell suspension, then fixed the cells in ice‐cold 75% ethanol. The fixed cells were stored at ‐20°C overnight. The next day, we centrifuged the cells at 1,000 × g for 5 min to form a pellet and carefully removed the supernatant. We washed the pellet twice with precooled PBS, then resuspended each sample in 0.5 mL of PI/RNase Staining Buffer (BD Pharmingen, USA). After gentle mixing, the cells were incubated at 37°C for 30 min in the dark. Finally, we analyzed the cells using a FACSCalibur flow cytometer (BD Biosciences).

For the apoptosis detection assay, we followed the same experimental conditions and exposure times used in the cell cycle analysis. After 48 h of treatment, we collected the cells and washed them twice with precooled PBS. The cells were resuspended in 1X Binding Buffer at a concentration of 1 × 10^6^ cells/ml, then stained with Annexin V‐kFluor647 and 100 nm SYTOX, according to the manufacturer's instructions (KeyGEN Biotech, China). We quantified apoptotic cells by determining the percentage of cells positive for Annexin V. Flow cytometric analysis was performed using FlowJo Software (BD Biosciences).

For the ROS detection assay, we preprocessed the cells following the same steps as in the apoptosis assay. The cells were then incubated with the ROS probe DCFH‐DA (Beyotime, #S0033S, China) at 37°C for 30 min in the dark prior to analysis.

### 2.8 In Vivo Experiments

All mouse experiments were approved by the Institutional Animal Care and Use Committee of Peking University First Hospital (No. J2025057). Balb/c‐Nu mice were maintained under a 12‐h light/dark cycle with unrestricted access to food and water. MIA PaCa‐2 cells (5 × 10^6^) in 100 µl of PBS mixed with Matrigel (1:1) were subcutaneously injected into the unilateral axillary region of the mice. Tumor dimensions were assessed every three days using manual palpation and digital calipers. Once the tumors reached a volume of 100 mm^3^, the mice were randomly divided into six groups (10 mice per group) based on tumor volume: (1) Vehicle; (2) IN10018 (25 mg/kg); (3) IN10018 (50 mg/kg); (4) Gemcitabine (50 mg/kg); (5) IN10018 (25 mg/kg) combined with gemcitabine (50 mg/kg); (6) IN10018 (50 mg/kg) combined with gemcitabine (50 mg/kg). IN10018 was administered orally via gavage daily, while gemcitabine was delivered by intraperitoneal injection. Tumor dimensions and volumes (calculated as 1/2 × length × width^2^) as well as body weights were recorded every three days. At the indicated time points, tumor sizes were measured, and the mice were euthanized. The excised tumors were stored in liquid nitrogen.

To establish an orthotopic KPC‐Luc pancreatic cancer mouse model, we housed C57BL/6J mice under conditions identical to those used for Balb/c‐Nu mice. We injected KPC cells (2 × 10^5^) in 20 µl of PBS mixed with Matrigel (1:1) into the pancreas of mice through a left upper abdominal incision. We monitored orthotopic tumor growth using luciferase signal imaging on an IVIS Spectrum. We administered D‐Luciferin (150 mg/kg; PerkinElmer, USA) via intraperitoneal injection 10 min before imaging. We acquired the signal with open filters and small binning to expose the left flank. Tumor burden was assessed based on total flux. We initiated treatments once tumors were both palpable and visible through IVIS imaging, starting 7 days after tumor inoculation. Mice were randomly assigned to four groups (6 mice per group): (1) Vehicle; (2) IN10018 (25 mg/kg); (3) Gemcitabine (25 mg/kg); (4) Combination (IN10018 and gemcitabine).

### 2.9 RNA‐Seq and Enrichment Analysis

We extracted total RNA according to the manufacturer's instructions for TRIzol reagent (Thermo Scientific). We prepared DNA libraries using the NEBNext Ultra Directional RNA Library Prep Kit for Illumina (New England Biolabs). We conducted transcriptome RNA sequencing (RNA‐Seq) using the Illumina HiSeq 2500 sequencer. We performed differential expression analysis of RNA‐Seq data between any two groups with DESeq2 [15]. We considered genes with Benjamini‐Hochberg–adjusted *p* < 0.05 and absolute log_2_[fold changes] >1 as differentially expressed. We conducted Gene Ontology (GO) and pathway enrichment analyses, including Kyoto Encyclopedia of Genes and Genomes (KEGG), for the differentially expressed genes using the R package clusterProfiler, with a significance threshold of *p* < 0.05 [16]. We visualized the data using the ggplot2 R package (https://ggplot2.tidyverse.org/).

### 2.10 Western Blot Analysis

We lysed cells on ice for 30 min using cell lysis buffer (Beyotime, China) supplemented with a protease inhibitor cocktail (Roche, USA). We centrifuged the lysates at 4°C (12,000 × g, 10 min) and separated the proteins by SDS‐PAGE (Biotides, China), transferring them to PVDF membranes (Bio‐Rad, USA). We blocked the membranes with 5% skim milk powder in TBST and incubated them with primary antibodies against SLC7A11 (Abclonal, #A2413, China), GPX4 (Abclonal, #A11243, China), and β‐actin (MBL, #PM053, Japan) at 4°C overnight. After incubation, we washed the membranes with TBST for 5 min (three times) and then incubated them with HRP‐conjugated goat anti‐rabbit IgG (H + L) (Earthox, #E030120, USA) or goat anti‐mouse IgG (H + L) (Earthox, #E030110) for 40 min. We developed the membranes using ECL substrate (Millipore, #WBULS0500, USA).

### 2.11 Histopathology, Immunohistochemistry of Paraffin Samples

We fixed murine stomach tissue overnight in 4% formalin, then transferred it to 70% ethanol for preservation. We subsequently embedded the samples in paraffin. We obtained sections with a thickness of 5 µm using a sliding microtome. To stain for Ki67, Sirius Red, Collagen‐I, and Fibronectin‐1, we performed heat‐induced antigen retrieval for 10 min using sodium citrate buffer. We achieved direct chromogenic visualization using the Liquid DAB Plus substrate reagent.

### 2.12 Analysis of Immune Cell Populations and Phenotypes

We harvested tumors and cut them into small pieces in cold PBS. After mechanical disruption, we incubated the tumor pieces in 5 mL of digestion solution (RPMI 1640 with 1.5% FBS, 0.5 mg/ml collagenase IV, 0.5 mg/ml collagenase I, and 0.04 mg/ml *DNase* I) for 20 min at 37°C while shaking at 200 rpm. We vortexed the suspension for 30 s before filtering it through a 70 µm cell strainer. We collected single‐cell suspensions in PBS for flow cytometry analysis. Detailed information about the antibodies used is provided in Table S1.

## 3 Results

### 3.1 Focal Adhesion Signal Might Contribute to Chemoresistance of PDAC

In order to elucidate the mechanisms underlying chemoresistance in pancreatic cancer, we herein focused on the evolution of drug resistance following chemotherapy and the role of the tumor microenvironment (TME). Both single‐cell RNA sequencing (scRNA‐seq) and bulk RNA sequencing analyses using data from the Gene Expression Omnibus (GEO) database were employed to identify hub genes and signaling pathways associated with chemoresistance in PDAC, we conducted both single‐cell RNA sequencing (scRNA‐seq) and bulk RNA sequencing analyses using data from the Gene Expression Omnibus (GEO) database. For the scRNA‐seq dataset (GSE205013), we selected four chemotherapy‐treated samples and four naïve samples based on specific inclusion criteria: all samples were obtained exclusively from in situ pancreatic tissue following surgical excision. We excluded samples with particular genomic variations. We performed gene filtering, normalization, and principal component analysis. After filtering out low‐quality cells and genes and removing doublets, we generated a gene‐cell matrix comprising 56,870 cells and 25,027 genes using the Seurat [17] package in R software. Pre‐ and post‐quality control records are presented in Figure S1A–C.

**FIGURE 1:**
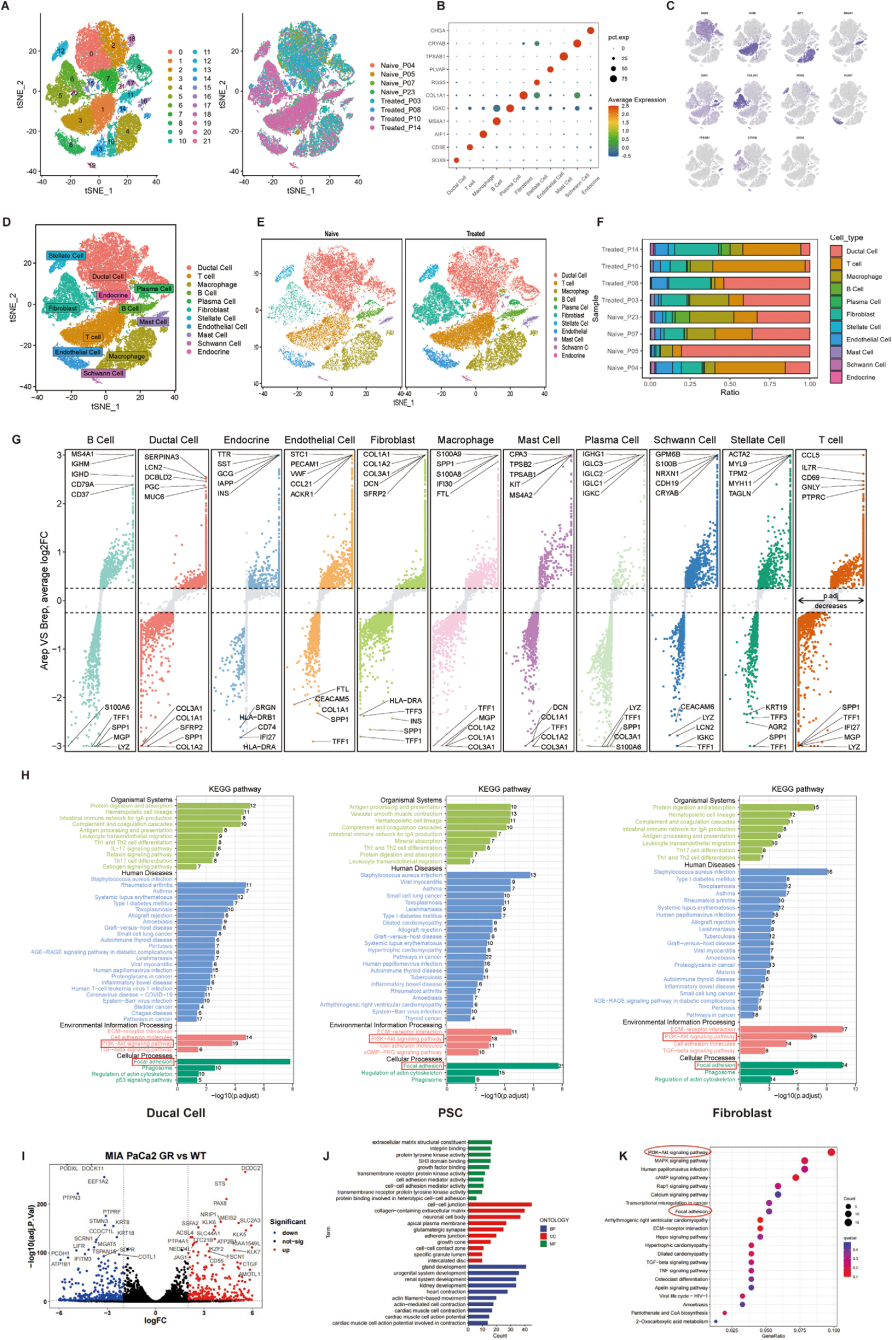
Activation of focal adhesions and PI3K‐Akt signaling accompanying chemotherapy and chemoresistance for PDAC. (A–H) scRNA sequencing analysis identifying the upregulated signals based on treatment. (A) t‐SNE plot showing main cell clusters in the treated and naïve tissues; (B) Dot plots showing the expression levels of key marker genes for each cell type; (C) Feature plots showing the expression distribution levels of marker genes; t‐SNE plot showing a summary of each cell type (D) and separated (E) based on the treatment; (F) Proportion of cells in each sample; (G) Volcano plots showing the top‐5 expression genes in each cell type; (H) KEGG enrichment analysis showing the significant enriched pathways in ductal cells, pancreatic stellate cells, and fibroblast cells; (I–K) Bulk‐RNA sequencing analysis identifying the upregulated pathways based on gemcitabine resistance. (I) Volcano plots showing the top‐20 expression genes; GO (J) and KEGG (K) enrichment analysis showing significantly enriched pathways.

We delineated the cell atlas using T‐distributed Stochastic Neighbor Embedding (t‐SNE) to visualize and segregate cells into distinct clusters based on their transcript profiles (Figure 1A). We identified a total of 21 original clusters, and Figure S1D presents the top five cell‐specific genes for each cluster. We then labeled these clusters with their corresponding cell types by cross‐referencing the cluster‐specific genes with established markers from previous studies [18, 19]. In total, we identified 11 known cell types: ductal cells (SOX9), T cells (CD3E), macrophages (AIF1), B cells (MS4A1), plasma cells (IGKC), fibroblasts (COL1A1), stellate cells (RGS5), endothelial cells (PLVAP), mast cells (TPSAB1), Schwann cells (CRYAB), and endocrine cells (CHGA) (Figure 1B,D). We demonstrated all cell types from treated and naïve samples, respectively (Figure 1E). We calculated the composition ratios of each cell type based on the sample sources (Figure 1F). Differential analysis was performed by cell type, with the top five up‐ and down‐regulated genes illustrated in volcano plots (Figure 1G). Following this, we conducted KEGG enrichment analysis for each cell type, revealing that focal adhesion signaling was the most significantly enriched pathway in ductal cells, stellate cells, and fibroblasts (Figure 1H). This finding suggests that the upregulation of focal adhesion signaling may contribute to the development of chemoresistance in pancreatic cancer.

To validate our findings from scRNA‐seq, we analyzed bulk RNA‐seq data from GEO datasets, specifically comparing gemcitabine‐resistant MIA PaCa‐2 cell lines with wild‐type cell lines (GSE148200). We performed differential analysis (Figure 1I). GO analysis revealed significant enrichment in pathways related to the extracellular matrix and collagen (Figure 1J). Additionally, we found that focal adhesion and PI3K‐Akt signaling pathways were significantly enriched (Figure 1K). Collectively, these results suggest that inhibiting focal adhesion signaling could reduce chemoresistance in PDAC and that the evolution of chemoresistance may be associated with the PI3K‐Akt signaling pathway.

### 3.2 FAK Inhibitor, IN10018, Cooperates With Gemcitabine and Enhances Its Sensitivity for PDAC In Vitro

**FIGURE 2:**
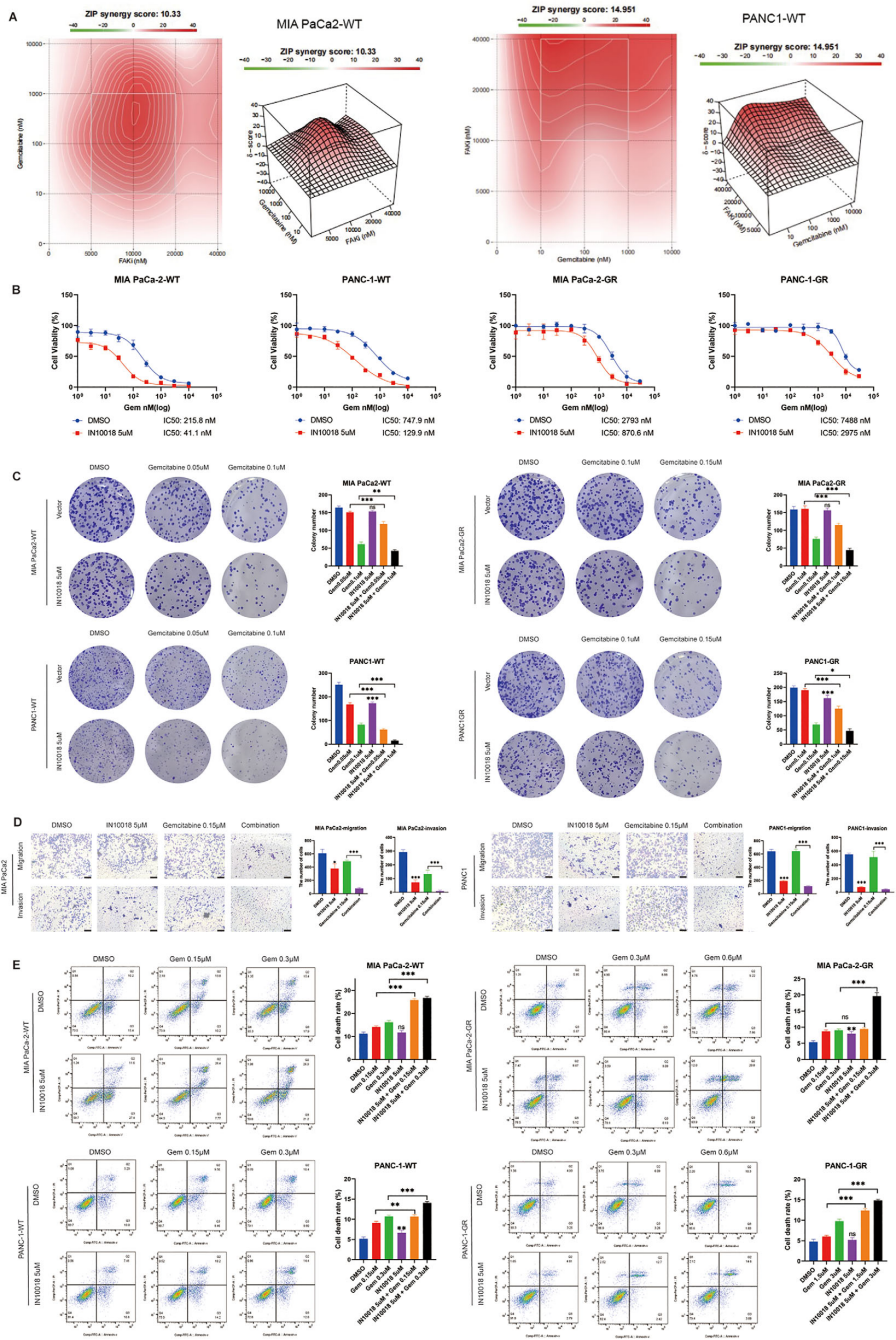
Focal adhesion kinase inhibitor (IN10018) sensitizing chemotherapy in human PDAC WT and GR cell lines in vitro. (A) Representative synergy models of FAKi and Gem for various PDAC cell lines. (B) Dose‐response curves for Gem or combination therapy with IN10018 in PDAC cell lines. Gem was evaluated in 3 serially diluted doses. Each dose was analyzed in technical triplicates for each biological replicate. Relative cell numbers were determined using CCK‐8 kits via enzyme calibration. (C) Crystal violet staining of colonies after 2 weeks after treatment with DMSO, IN10018 (5 µm), Gem (0.05 and 0.1 µm for WT, 0.1 and 0.15 µm for GR), and combination therapy. (D) Representative images and statistical results of migration and invasion after 48 h of the indicated treatments (bar: 100 µm). (E) Apoptosis assays and statistical results after 48 h of the indicated treatments.

To validate our hypothesis, we utilized a novel focal adhesion kinase inhibitor, IN10018, in combination with the first‐line chemotherapeutic agent gemcitabine in subsequent experiments. We employed SynergyFinder [20] (https://synergyfinder.fimm.fi/) to accurately assess the synergistic effects and calculate synergy scores for the IN10018 and gemcitabine combination. Evidence suggests that the MIA PaCa‐2 and Panc‐1 cell lines exhibit relatively higher resistance to gemcitabine among PDAC cell lines; thus, we selected these two cell lines for further investigation in the synergistic experiments. Consistent with our hypothesis, the combination of IN10018 and gemcitabine demonstrated moderate synergistic effects in both cell lines (Figure 2A).

To confirm our findings further, we generated dose‐response curves to calculate the 50% inhibitory concentration (IC_50_) of gemcitabine for both monotherapy and combination groups. We also included GR MIA PaCa‐2 and PANC‐1 cell lines, along with their wild‐type (WT) counterparts, in subsequent phenotypic experiments. The results indicated that IN10018 enhanced the sensitivity of PDAC‐WT cells to gemcitabine and reduced resistance in PDAC‐GR cells (Figure 2B).

To elucidate the pharmacological potency of the IN10018 and gemcitabine combination, we conducted a colony formation assay (Figure 2C). The results consistently demonstrated enhanced sensitivity in WT cell lines and reduced resistance in GR cell lines. Also, to excluding off‐target effect, knockdown of FAK using siRNA enhanced the sensitivity of gemcitabine for pancreatic cancer (Figure S2). To further investigate the pharmacological effects on PDAC invasiveness, we conducted migration and invasion assays. The results indicated that the combination of IN10018 and gemcitabine significantly inhibited cell invasiveness, particularly compared to gemcitabine monotherapy (Figure 2D). Additionally, cell scratch assays corroborated these findings, revealing consistent trends in both WT and GR cell lines (Figure S3A,B).

We also performed flow cytometric analyses to assess apoptosis and cell cycle progression. The apoptosis assays demonstrated that the combination of IN10018 and gemcitabine produced a moderate synergistic effect in both WT and GR cell lines (Figure 2E). In contrast, the cell cycle assays did not reveal significant differences in either WT or GR cell lines (Figure S3C). These findings suggest that IN10018 primarily enhances sensitivity to gemcitabine‐induced apoptosis rather than altering cell cycle dynamics.

In summary, the combination of gemcitabine and focal adhesion kinase inhibition exhibits a significant synergistic effect on chemosensitivity, primarily due to the induction of apoptosis.

### 3.3 Combination of IN10018 and Gem Shows Potent Antitumor Effects In Vivo

**FIGURE 3:**
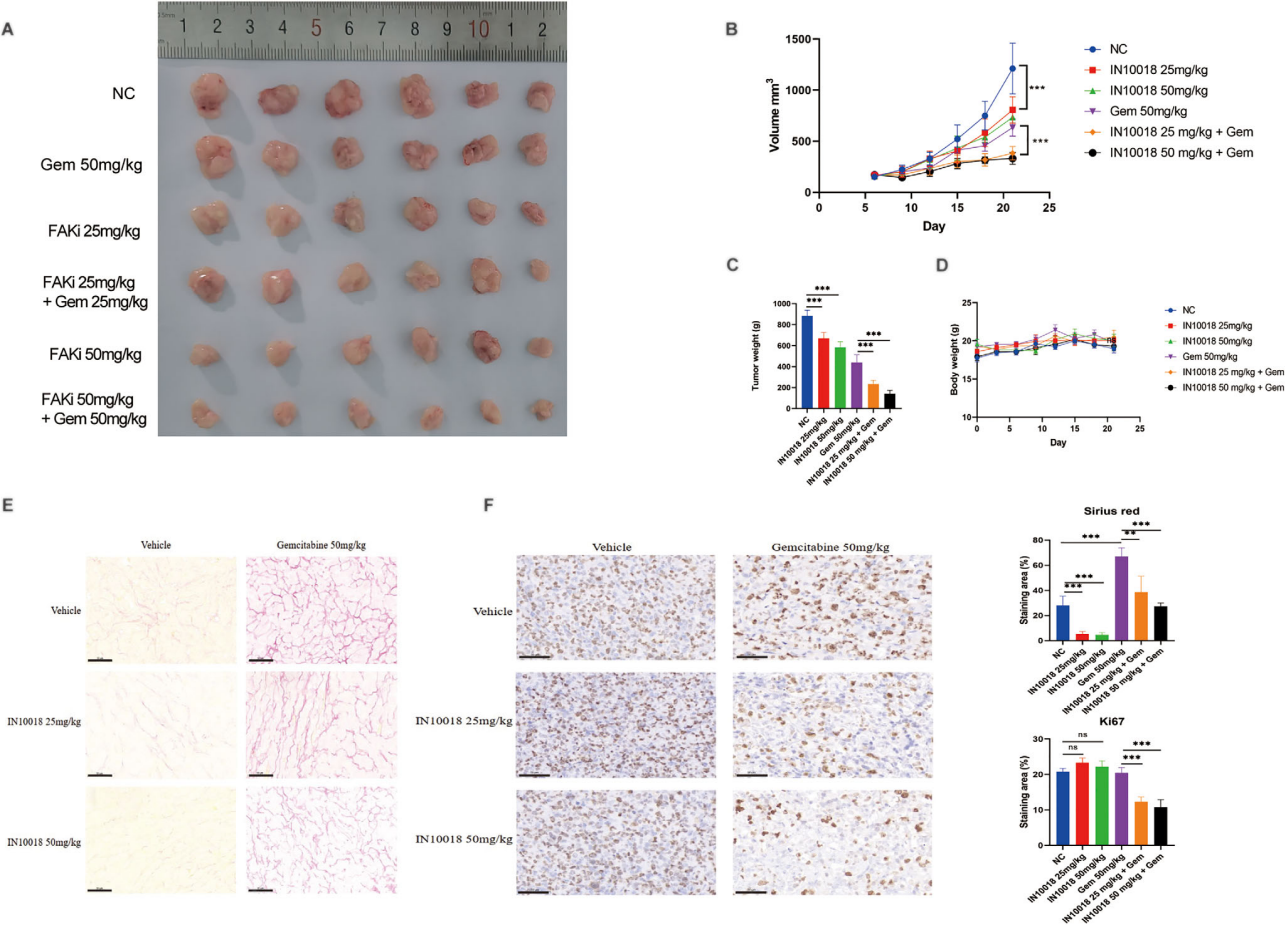
IN10018 enhances Gem sensitivity for PDAC in vivo. (A) Subcutaneous tumor model showing that combination therapy of IN10018 and Gem improved antitumor efficacy. (B) Tumor volumes measured and presented as mean ± SD (*n* = 10/group). (C) Histogram showing the mean ± SD of tumor weights from indicated groups at the endpoint. (D) Line chart showing changes in mouse weight of the indicated groups. (E,F) Representative images showing immunohistological evaluation of the extracellular matrix via Sinus red staining (E) and cellular proliferation via Ki‐67 staining (F) (bar: 50 µm).

To evaluate the efficacy of IN10018 and gemcitabine in vivo, we employed a CDX model using the PDAC cell line MIA PaCa‐2 (Figure 3A). The combination treatment significantly restricted tumor growth in the MIA PaCa‐2 xenograft model compared to either monotherapy. Furthermore, the combination treatment led to a notable reduction in tumor weight (Figure 3B,C). Throughout the dosing period, all mice tolerated the treatment well, with no statistically significant differences in body weight among the groups (Figure 3D). Additionally, immunohistochemical (IHC) analysis of tumors collected at the treatment endpoints demonstrated marked suppression of Ki67 expression in the combination‐treated group (Figure 3E). Notably, while gemcitabine monotherapy increased stromal collagen intensity, the combination of IN10018 reversed these changes in the TME, which could otherwise promote tumor growth and chemoresistance (Figure 3F). Collectively, these results highlight the in vivo antitumor efficacy of the combination treatment with IN10018 and gemcitabine for PDAC.

### 3.4 IN10018 Regulates Chemosensitivity of PDAC Cells Through SLC7A11

**FIGURE 4:**
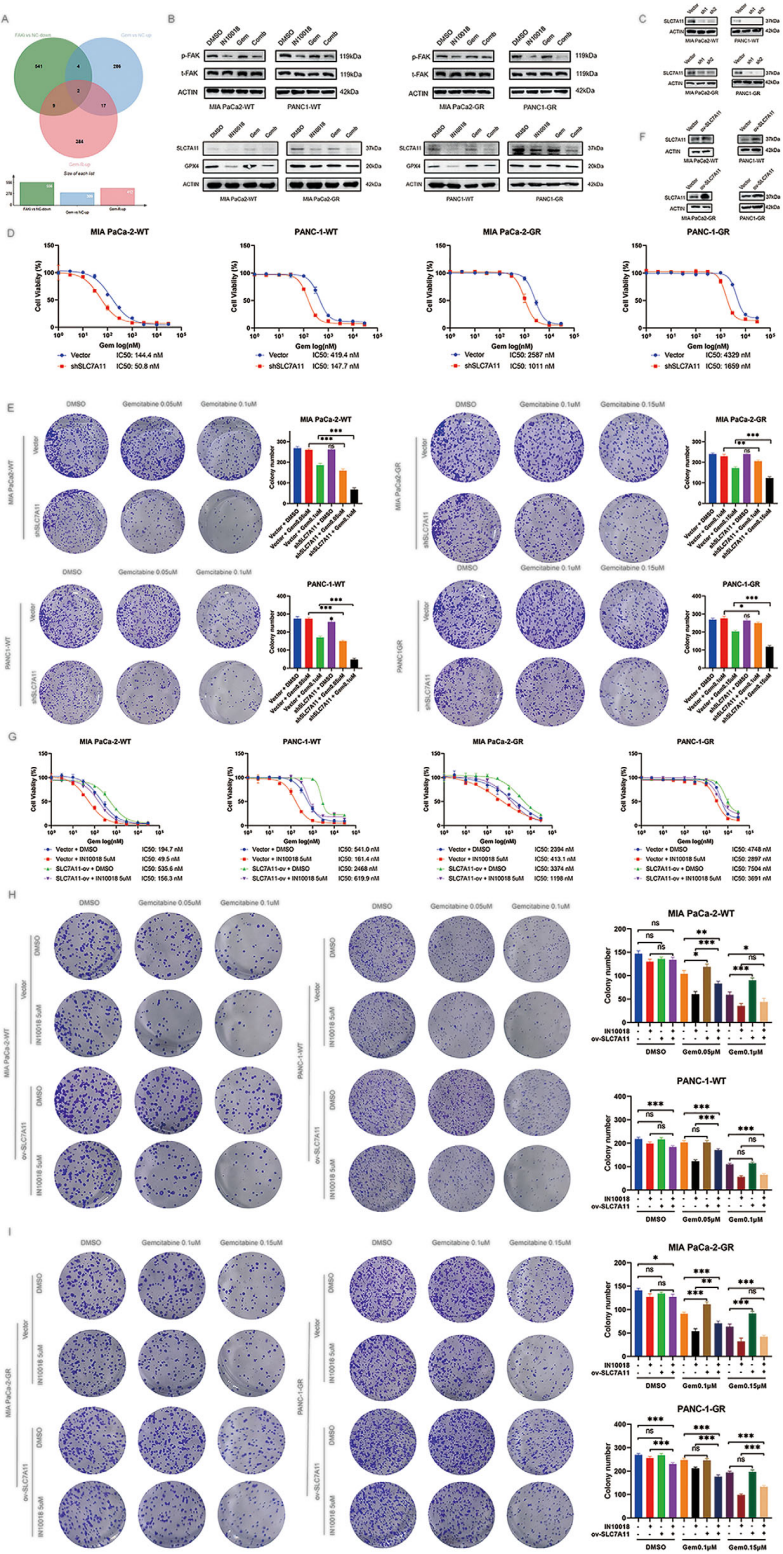
IN10018 regulates chemosensitivity of PDAC cells through SLC7A11. (A) Venn diagram showing the intersected differentially expressed genes of three gene sets (upregulating DEGs of GR vs. WT and Gem treated vs. NC; downregulating DEGs of IN10018 treated vs. NC). (B) IN10018 affects SLC7A11 and GPX4 in WT and GR cells. (C) Protein levels of SLC7A11 in PDAC cell lines transfected with shRNAs were detected by Western blot. (D) Dose‐response curves of Gem or combination therapy with shSLC7A11 in WT and GR cell lines. (E) Crystal violet staining of colonies after 2 weeks of treatment with vector, shSLC7A11, Gem (0.05 and 0.1 µm for WT, 0.1 and 0.15 µm for GR), and combination therapy. (F) Protein levels of SLC7A11 in PDAC cell lines transfected with lentiviral plasmid overexpressing SLC7A11 were detected by Western blot. (G‐H) Rescue experiments of IN10018 and SLC7A11 were designed with dose‐response curves (G) and crystal violet staining of colonies (H) in WT and GR cell lines.

To elucidate the molecular mechanisms by which IN10018 enhances gemcitabine sensitivity in PDAC cells, we conducted transcriptomic profiling of MIA PaCa‐2 cells under four treatment conditions: DMSO, IN10018, gemcitabine, and their combination. By comparing differentially expressed genes (DEGs) between WT and GR cells in PDAC GEO datasets, we identified SLC7A11 as a potential target of IN10018 (Figure 4A).

We performed Western blot analysis to confirm the molecular changes induced by IN10018. The results indicated that IN10018 inhibited SLC7A11 expression in both WT and GR PDAC cell lines. Furthermore, IN10018 also reduced the expression of the ferroptosis‐associated gene GPX4 (Figure 4B), suggesting that IN10018 may enhance chemosensitivity by promoting ferroptosis. Subsequently, we established SLC7A11 knockdown WT and GR cell lines and confirmed the efficiency of the knockdown using Western blot analysis (Figure 4C). SLC7A11 knockdown increased the chemosensitivity of WT cell lines and diminished the chemoresistance of GR cell lines (Figure 4D). Consistent with these findings, the colony formation assay showed similar trends (Figure 4E).

To verify the molecular regulatory relationship between FAK (the target of IN10018) and SLC7A11, we conducted a rescue experiment to determine whether SLC7A11 overexpression could reverse the inhibitory effects of IN10018. We established SLC7A11 overexpression WT and GR cell lines and confirmed the overexpression efficiency via Western blot (Figure 4F). Chemosensitivity curves demonstrated that SLC7A11 overexpression increased chemoresistance in both WT and GR PDAC cell lines, effectively reversing the antitumor effects of IN10018 (Figure 4G). Similarly, the colony formation assay produced consistent results, further supporting these findings (Figure 4H,I).

Furthermore, based on pancreatic cancer specimens previously collected at our institution, we selected cases with complete baseline and follow‐up information to construct tissue microarrays. Immunofluorescence staining was performed to assess the expression of p‐FAK and SLC7A11. The results showed that, compared with non‐chemotherapy samples, the levels of both p‐FAK and SLC7A11 were significantly upregulated in pancreatic cancer tissues after neoadjuvant chemotherapy, and their expression exhibited a strong positive correlation (Figure S4).

### 3.5 IN10018 Promotes SLC7A11‐Mediated Ferroptosis Through PI3K‐Akt Signal

**FIGURE 5:**
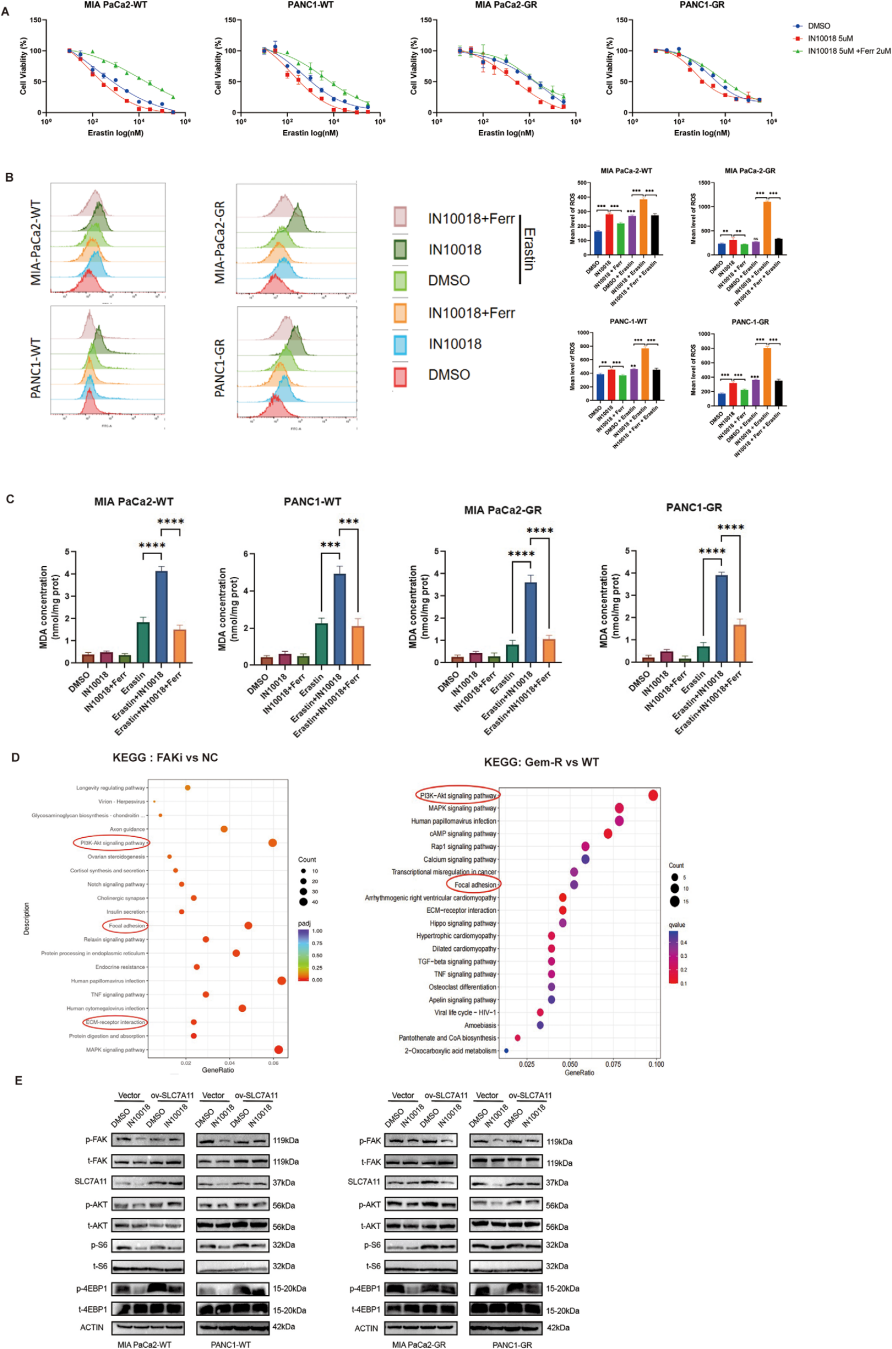
IN10018 promotes SLC7A11‐mediated ferroptosis through PI3K‐Akt signal. Recue experiments showing IN10018 regulates ferroptosis of WT and GR cell lines with sensitivity to ferroptosis inducer Erastin (A), intracellular ROS (B), and MDA (C) levels. (D) KEGG analysis showing enrichment of PI3K‐Akt and focal adhesion signaling in IN10018 vs. NC and Gem‐resistance vs. WT. (E) Protein levels of p‐FAK, t‐FAK, SLC7A11, p‐Akt, t‐Akt, p‐S6, t‐S6, p‐4EBP1, and t‐4EBP1 were measured by Western blot analysis in each cell line. β‐ACTIN was used as a loading control.

In recent years, SLC7A11 has been frequently implicated in tumor proliferation and chemoresistance by inhibiting ferroptosis [21, 22]. To investigate whether IN10018 promotes ferroptosis via SLC7A11, we conducted rescue assays. IN10018 enhanced the sensitivity of both WT and GR cells to ferroptosis inducers, such as Erastin, an effect that was attenuated by ferroptosis inhibitors (Figure 5A). Furthermore, IN10018 significantly increased intracellular levels of reactive oxygen species (ROS) (Figure 5B) and malondialdehyde (MDA) (Figure 5C) in response to ferroptosis inducers, while the ferroptosis inhibitor Fer‐1 effectively reversed these effects. These results indicate that IN10018 enhances sensitivity to ferroptosis in both WT and GR PDAC cells.

To elucidate the mechanism by which IN10018 enhances chemosensitivity, we performed KEGG pathway analysis based on differentially expressed genes between IN10018‐treated and control (downregulated) groups, as well as between gemcitabine‐resistant and wild‐type (upregulated) cells. The analysis revealed that the PI3K‐Akt signaling pathway was downregulated by IN10018, while it was upregulated in gemcitabine‐resistant cells (Figure 5D). These findings suggest that the PI3K‐Akt pathway may serve as a critical mediator of IN10018's effects in enhancing chemosensitivity and mitigating chemoresistance in PDAC. Consistent with this hypothesis, Western blot analysis confirmed that IN10018 treatment aligned with our expectations regarding modulation of the PI3K‐Akt pathway (Figure 5E). Also, overexpression of AKT could rescue the downregulation of SLC7A11 by IN10018, which illustrated the molecular mechanism of IN10018 regulating SLC7A11 through the PI3K‐Akt signaling pathway (Figure S5).

### 3.6 IN10018 Restricts Abundance of Mesenchyme and Reconstructs the TIME

**FIGURE 6:**
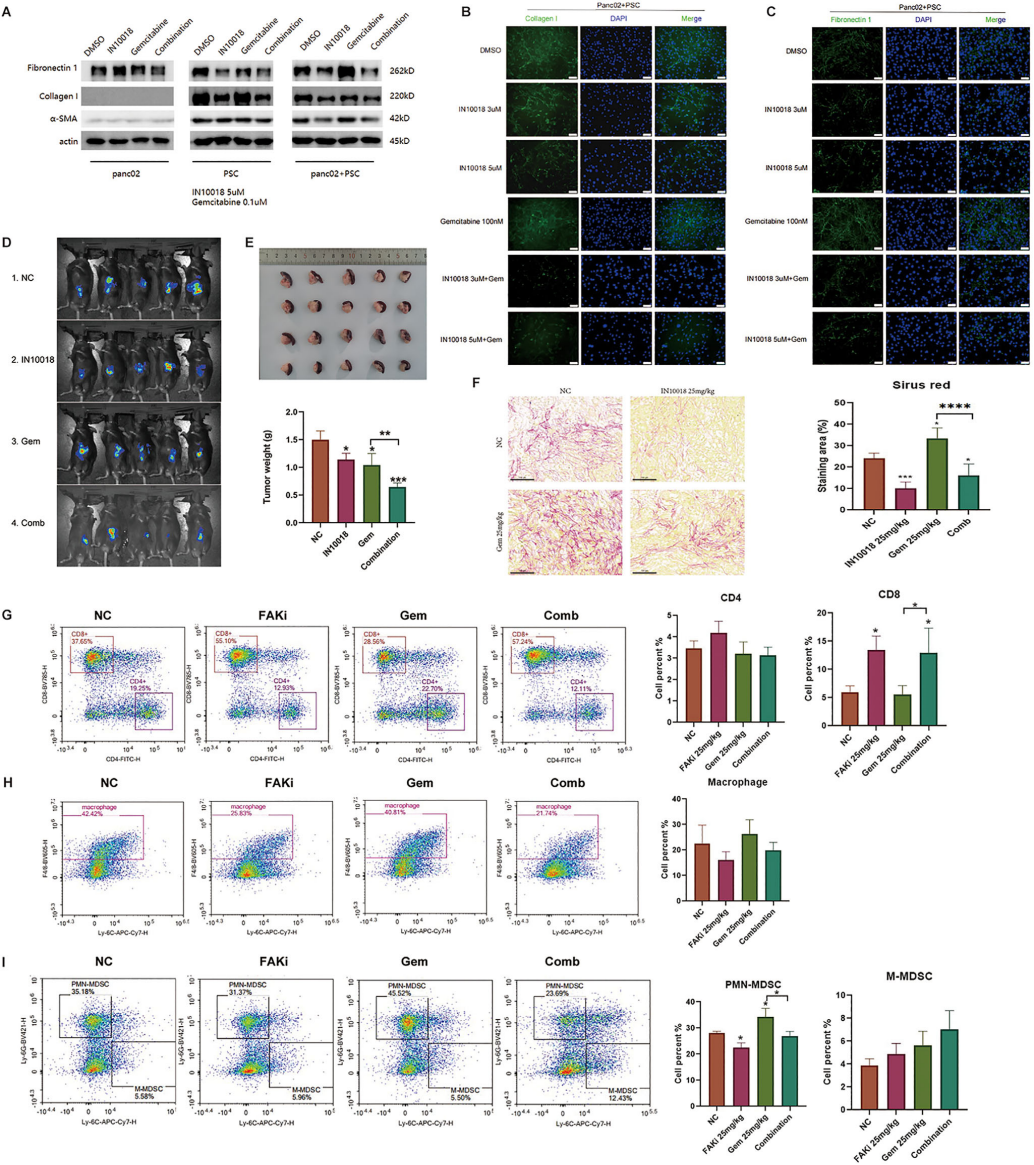
IN10018 restricts the abundance of mesenchyme and reconstructs the TIME. (A) Western blot measuring protein levels of Fibronectin‐1, Collagen‐1, and α‐SMA in panc02, PSC, and coculture cell lines after treatment with DMSO, IN10018 (5 µm), Gem (0.1 µm), and combination therapy. (B,C) Immunofluorescence staining measuring protein levels of Fibronectin‐1, Collagen‐1 in panc02, PSC, and coculture cell lines after treatment with DMSO, IN10018 (3 and 5 µm), Gem (0.1 µm), and combination therapy (bar: 100 µm). (D) Orthotopic xenograft model measured by fluorescence imaging in the indicated groups of DMSO, IN10018 (25 mg/kg), Gem (25 mg/kg), and combination therapy. (E) Tumors derived from an orthotopic xenograft model showing the combination therapy of IN10018 and Gem improves antitumor efficacy. (F) Representative images showing immunohistological evaluation of the extracellular matrix via Sinus red staining (bar: 100 µm). (G–I) Representative flow cytometry plot and percentage of CD8^+^, CD4^+^ T cells, macrophages, PMN‐MDSC, and M‐MDSC.

To evaluate the impact of IN10018 on the TME, particularly the mesenchymal and immune components, we conducted in vitro co‐culture assays of PDAC cells and pancreatic stellate cells (PSC), along with in vivo orthotopic xenograft models. In mono‐culture assays, western blot analysis showed that IN10018 reduced Fibronectin‐1 levels in both PDAC and PSC cells, as well as Collagen‐I levels in PSC cells. In co‐culture assays, gemcitabine treatment increased Fibronectin‐1 levels, a trend that IN10018 inhibited. Furthermore, IN10018 decreased α‐SMA expression, indicating reduced activation of PSCs and fibroblasts (Figure 6A). Immunofluorescence staining supported these results, revealing a dose‐dependent effect of IN10018 (Figure 6B,C).

Results from the orthotopic xenograft model demonstrated a synergistic effect of IN10018 and gemcitabine, as evidenced by fluorescence imaging (Figure 6D) and tumor weight measurements (Figure 6E). Consistent with findings from the nude mice model, gemcitabine monotherapy increased collagen intensity, while the combination of IN10018 and gemcitabine significantly diminished this effect (Figure 6F).

We then digested tumor tissues and isolated immune cells for flow cytometric analysis to further investigate the tumor microenvironment. IN10018 significantly increased CD8^+^ T cell levels (Figure 6G), while we observed a notable trend toward reduced macrophage levels (Figure 6H). In terms of myeloid‐derived suppressor cells (MDSCs), gemcitabine led to an increase in polymorphonuclear neutrophil (PMN‐MDSC) levels, which IN10018 countered (Figure 6I). Collectively, these findings indicate that IN10018 reduces the abundance of mesenchymal components, such as collagen‐I and Fibronectin‐I, and positively modulates the tumor immune microenvironment by enhancing CD8^+^ T cell infiltration and inhibiting PMN‐MDSCs.

## 4 Discussion

Gemcitabine monotherapy, often combined with other agents, serves as the first‐line treatment for pancreatic cancer in adjuvant, post‐operative, and palliative settings [23]. However, both natural and acquired chemoresistance significantly limit its efficacy, with objective response rates for gemcitabine monotherapy ranging from 8% to 11% [7]. Despite the development of numerous novel targeted therapies and inhibitors over the past decade, which have produced only partial improvements in prognosis when combined with gemcitabine, the desired synergistic effects have not yet been achieved. Therefore, addressing and overcoming chemoresistance remains a critical strategy for enhancing chemotherapy efficacy.

In our primary target screening for key pathways associated with chemoresistance, we investigated potential targets in pancreatic cancer using scRNA‐seq and bulk RNA sequencing (bulk‐seq) analyses. By comparing chemotherapy‐treated versus naïve samples and chemoresistant versus wild‐type samples, we identified the focal adhesion signaling pathway as a potential critical target. FAK was enriched in various cell types, including ductal cells, fibroblasts, and pancreatic stellate cells, suggesting that focal adhesion signaling may contribute to chemoresistance by enhancing the tumor microenvironment. Wu et al. [24] were the first to demonstrate that FAK phosphorylation contributes to intrinsic chemoresistance to gemcitabine in pancreatic cancer cell lines. Similarly, Jiang et al. [13] showed that FAK inhibition enhances immune surveillance by counteracting the fibrotic and immunosuppressive characteristics of the PDAC tumor microenvironment, thereby improving tumor responsiveness to immunotherapy. In this study, we further found that, relative to non‐chemotherapy patients, the FAK pathway was further upregulated in patients who had undergone chemotherapy, suggesting that it may also be involved in the chemotherapeutic evolutionary process of pancreatic cancer. This observation is consistent with previous findings. Based on these findings, we hypothesize that FAK inhibitors may mitigate chemoresistance in PDAC by downregulating focal adhesion signaling.

To validate our hypothesis, we employed a novel FAK inhibitor (IN10018) and assessed its effects in combination with gemcitabine on both wild‐type and gemcitabine‐resistant PDAC cell lines in in vitro and in vivo models. Our results demonstrated that IN10018 synergized effectively with gemcitabine, enhancing apoptosis in PDAC cells. Additionally, both monotherapy and combination treatment with IN10018 significantly inhibited cell migration and invasion. In vivo, gemcitabine monotherapy increased stromal components in the tumor microenvironment, including collagen and fibronectin, which may contribute to chemoresistance. However, the combination of IN10018 and gemcitabine reversed this trend, potentially mitigating chemoresistance. For other FAK inhibitors, VS‐4718 were reported as one of FAK inhibitors, together with GSK2256098, VS‐6062, VS‐6063, and BI853520, which were reported to be used in clinical trials. However, the effect of reversing gemcitabine chemoresistance has never been reported in pancreatic cancer [25].

FAKi have been extensively reported to exert anti‐tumor effects through multiple downstream molecules and pathways. In this study, we aimed to identify additional mechanisms by examining the impact of FAKi on gemcitabine chemoresistance. We intersected the upregulated genes in gemcitabine‐resistant PDAC cells with the downregulated genes following IN10018 treatment, indicating that SLC7A11 may be a key downstream target.

SLC7A11 is a crucial component of the cystine/glutamate transporter, playing a significant role in cellular metabolism and the regulation of ferroptosis [21]. Ferroptosis, a regulated form of cell death driven by metal‐catalyzed lipid oxidation, is negatively regulated by SLC7A11 through its role in cystine absorption. This process supports the production of antioxidant enzymes and reduces harmful oxidative species [26]. Ferroptosis has been linked to chemoresistance in various cancers and is emerging as a potential target for combination therapies [26, 27]. In PDAC, SLC7A11 induces tumor‐selective ferroptosis and inhibits tumor growth in genetically engineered mice lacking SLC7A11 [28]. Despite these findings, the relationship between FAK and SLC7A11 remains unclear. Our study demonstrates that IN10018, a FAKi, reduces SLC7A11 levels, thereby promoting apoptosis in PDAC cells and enhancing sensitivity to gemcitabine while reversing resistance through ferroptosis pathways. The effects of IN10018 were mitigated by the ferroptosis inhibitor Fer‐1, further confirming the regulatory relationship between FAKi and ferroptosis.

Our findings on the TME corroborate previous studies [13], indicating that IN10018 reduces collagen intensity and promotes the infiltration of CD8^+^ T cells. Notably, we observed that gemcitabine treatment increased stromal components, including collagen and fibronectin, in co‐culture conditions with PDAC cells and fibroblasts. In contrast, IN10018 reversed these changes and further decreased stromal intensity. Previous research has linked various stromal components, such as laminin, collagen I, and fibronectin, to intrinsic chemoresistance in multiple cancers, including PDAC [28, 29]. Murphy et al. demonstrated that stromal stiffness correlates with PDAC chemoresistance [30]. Osipov et al. demonstrated that inhibition of FAK enhances the antitumor response of radiation therapy in pancreatic cancer through CD8+ T cells using a murine model, suggesting the potential application of IN10018 in clinical pancreatic cancer treatment [31]. These findings support our hypothesis that IN10018 enhances chemosensitivity and mitigates chemoresistance in PDAC by modulating the TME, specifically targeting collagen and fibronectin. Furthermore, the observed increase in immune cell infiltration may relate to alterations in stromal intensity and stiffness following FAKi treatment.

Emerging evidence has established ferroptosis as an immunogenic cell death modality. Lipid peroxidation and subsequent cell death lead to the release of damage‐associated molecular patterns (DAMPs), including HMGB1 and oxidized mitochondrial DNA (ox‐mtDNA), which activate the cGAS‐STING pathway and promote CD8^+^ T cell priming and tumor infiltration [32]. In our study, FAK inhibition‐induced downregulation of SLC7A11 triggered ferroptosis in tumor cells, accompanied by increased intratumoral CD8^+^ T cell accumulation. We propose that this immune infiltration is not a parallel event, but rather a direct consequence of ferroptosis‐initiated immunogenicity.

Within the tumor compartment, IFNγsecreted by infiltrating CD8^+^ T cells suppresses SLC7A11 expression and sustains ferroptosis in cancer cells via the JAK‐STAT1 axis, forming a positive feedback loop that amplifies both cell death and antitumor immunity [33, 34]. This T cell–ferroptosis crosstalk has been well documented in multiple cancer types and represents a core mechanism of immune‐mediated tumor suppression.

Concurrently, stromal remodeling—characterized by reduced collagen deposition and enhanced T cell infiltration—can be achieved by directly targeting CAF activation pathways such as FAK [35, 36]. Our data demonstrate that FAK inhibition not only suppresses SLC7A11 in tumor cells but also attenuates CAF‐mediated matrix stiffening, facilitating CD8^+^ T cell access. These two arms are not mutually exclusive; rather, they may cooperatively reinforce each other. In summary, our findings suggest that FAK inhibition acts as a dual trigger: it directly induces tumor ferroptosis while simultaneously remodeling the physical barrier imposed by CAFs. The resulting immunogenic niche and positive feedback loop between ferroptosis and CD8^+^ T cells may explain the potent and durable antitumor effects observed upon FAK inhibition. Future studies investigating whether IFNγdirectly reprograms CAF metabolism and SLC7A11 expression will further clarify the full spectrum of this ferroptosis–immunity–stroma axis.

Despite our promising findings, several limitations exist in this study. First, we only validated the relationship between FAK and SLC7A11 through rescue assays, without further exploring the detailed regulatory mechanisms involved. Second, while we demonstrated the effects of IN10018 on the TME, we did not conduct rescue assays to investigate the underlying intrinsic mechanisms. Third, our validation of the synergistic effects of IN10018 and gemcitabine was confined to PDAC cell lines derived from humans and mice. To gain a more comprehensive understanding of IN10018's potential, future studies should incorporate additional experimental models, such as patient‐derived xenografts (PDXs) and patient‐derived organoids (PDOs), which more accurately mimic the clinical environment and can better predict real‐world therapeutic outcomes.

In this study, we conducted a thorough preclinical evaluation of the combination of the FAK inhibitor (IN10018) and gemcitabine as a first‐line therapy for PDAC. We explored a novel mechanism that reduces chemoresistance, including the regulation of ferroptosis and the reversal of stromal conditions following chemotherapy.

## Author Contributions

Y.W, H.Y, Y.Z and Y.M developed the study concept and were responsible for the study design. Y.W and J.Y analyzed the scRNA‐Seq and bulk‐seq data. Y.W., H.Y., and B.Z performed the experiments and wrote the manuscript, which was then edited by all co‐authors. K.Z and P.W provided technical guidance of experiments. Z.W, R.L, X.T, N.Z and Y.Y directed the study.

## Funding

This study was supported by the National Key Research and Development Program of China (2021YFA0909900, 2023YFC2413400), National Natural Science Foundation of China (NO. 82171722, 82271764, 82471772, 82588201, 82573723 and 82003187), Beijing Natural Science Foundation (L246015), National High Level Hospital Clinical Research Funding (Interdepartmental Research Project of Peking University First Hospital 2023IR23, 2024IR11), National High Level Hospital Clinical Research Funding (Scientific Research Seed Fund of Peking University First Hospital 2023SF47), National High Level Hospital Clinical Research Funding (Youth Clinical Research Project of Peking University First Hospital 2023YC06), National High Level Hospital Clinical Research Funding (Scientific Research Fund of Peking University First Hospital 2024XTZ10), “Star of Outlook” Scientific Research Project of Peking University First Hospital 2024XW02, Young Elite Scientists Sponsorship Program of the Beijing High Innovation Plan 20250797, Research and Translational Application of Clinical Characteristic Diagnosis and Treatment Techniques in the Capital (Z221100007422070), and also supported by the State Key Laboratory of Natural and Biomimetic Drugs.

## Conflicts of Interest

The authors declare no conflict of interest.

## Supporting information




**Supporting File**: advs75216‐sup‐0001‐SuppMat.docx.

## Data Availability

The data that support the findings of this study are available on request from the corresponding author. The data are not publicly available due to privacy or ethical restrictions.

## References

[1] R. L. Siegel , A. N. Giaquinto , and A. Jemal , “Cancer Statistics, 2024,” CA: A Cancer Journal for Clinicians 74, no. 1 (2024): 12–49, 10.3322/caac.21820. 38230766

[2] R. L. Siegel , K. D. Miller , H. E. Fuchs , and A. Jemal , “Cancer Statistics, 2022” CA: A Cancer Journal for Clinicians 72 (2022): 7–33, 10.3322/caac.21708. 35020204

[3] M. Espona‐Fiedler , C. Patthey , S. Lindblad , I. Sarró , and D. Öhlund , “Overcoming Therapy Resistance in Pancreatic Cancer: New Insights and Future Directions,” Biochemical Pharmacology 229 (2024): 116492. 39153553 10.1016/j.bcp.2024.116492

[4] J. Kleeff , M. Korc , M. Apte , et al., “Pancreatic Cancer,” Nature Reviews Disease Primers 2 (2016): 16022, 10.1038/nrdp.2016.22. 27158978

[5] T. Kamisawa , L. D. Wood , T. Itoi , and K. Takaori , “Pancreatic Cancer,” The Lancet 388 (2016): 73–85. 10.1016/S0140-6736(16)00141-0 26830752

[6] T. Conroy , F. Desseigne , M. Ychou , et al., “FOLFIRINOX versus gemcitabine for Metastatic Pancreatic Cancer,” New England Journal of Medicine 364, no. 19 (2011): 1817–1825. 21561347 10.1056/NEJMoa1011923

[7] D. D. Von Hoff , T. Ervin , F. P. Arena , et al., “Increased Survival in Pancreatic Cancer with Nab‐Paclitaxel plus Gemcitabine,” New England Journal of Medicine 369 (2013): 1691–1703. 24131140 10.1056/NEJMoa1304369 PMC4631139

[8] M. Dean , T. Fojo , and S. Bates , “Tumour Stem Cells and Drug Resistance,” Nature Reviews Cancer 5 (2005): 275–284. 15803154 10.1038/nrc1590

[9] L. Wei , H. Ye , G. Li , et al., “Cancer‐associated Fibroblasts Promote Progression and Gemcitabine Resistance via the SDF‐1/SATB‐1 Pathway in Pancreatic Cancer,” Cell Death & Disease 9 (2018): 1065. 30337520 10.1038/s41419-018-1104-x PMC6194073

[10] M. Ponz‐Sarvise , V. Corbo , H. Tiriac , et al., “Identification of Resistance Pathways Specific to Malignancy Using Organoid Models of Pancreatic Cancer,” Clinical Cancer Research 25 (2019): 6742–6755. 31492749 10.1158/1078-0432.CCR-19-1398 PMC6858952

[11] M. D. Schaller , C. A. Borgman , B. S. Cobb , R. R. Vines , A. B. Reynolds , and J. T. Parsons , “pp125FAK a Structurally Distinctive Protein‐tyrosine Kinase Associated with Focal Adhesions,” Proceedings of the National Academy of Sciences 89 (1992): 5192–5196. 10.1073/pnas.89.11.5192 PMC49256 1594631

[12] C. J. Diaz Osterman , D. Ozmadenci , E. G. Kleinschmidt , et al., “FAK Activity Sustains Intrinsic and Acquired Ovarian Cancer Resistance to Platinum Chemotherapy,” Elife 8 (2019): 47327. 10.7554/eLife.47327 PMC6721800 31478830

[13] H. Jiang , S. Hegde , B. L. Knolhoff , et al., “Targeting Focal Adhesion Kinase Renders Pancreatic Cancers Responsive to Checkpoint Immunotherapy,” Nature Medicine 22 (2016): 851–860. 10.1038/nm.4123 PMC4935930 27376576

[14] M. Feng , H. Xu , W. Zhou , and Y. Pan , “The BRD4 Inhibitor JQ1 Augments the Antitumor Efficacy of abemaciclib in Preclinical Models of Gastric Carcinoma,” Journal of Experimental & Clinical Cancer Research 42 (2023): 44. 36755269 10.1186/s13046-023-02615-2 PMC9909925

[15] M. I. Love , W. Huber , and S. Anders , “Moderated Estimation of Fold Change and Dispersion for RNA‐seq Data with DESeq2,” Genome Biology 15 (2014): 550. 25516281 10.1186/s13059-014-0550-8 PMC4302049

[16] G. Yu , L.‐G. Wang , Y. Han , and Q.‐Y. He , “clusterProfiler: an R Package for Comparing Biological Themes among Gene Clusters,” OMICS: A Journal of Integrative Biology 16 (2012): 284–287. 22455463 10.1089/omi.2011.0118 PMC3339379

[17] Y. Hao , S. Hao , E. Andersen‐Nissen , et al., “Integrated Analysis of Multimodal Single‐cell Data,” Cell 184 (2021): 3573–3587. 34062119 10.1016/j.cell.2021.04.048 PMC8238499

[18] K. Chen , Y. Wang , Y. Hou , et al., “Single Cell RNA‐seq Reveals the CCL5/SDC1 Receptor‐ligand Interaction between T Cells and Tumor Cells in Pancreatic Cancer,” Cancer Letters 545 (2022): 215834. 35917973 10.1016/j.canlet.2022.215834

[19] J. Peng , B.‐F. Sun , C.‐Y. Chen , et al., “Single‐cell RNA‐seq Highlights Intra‐tumoral Heterogeneity and Malignant Progression in Pancreatic Ductal Adenocarcinoma,” Cell Research 29 (2019): 725–738. 31273297 10.1038/s41422-019-0195-y PMC6796938

[20] A. Ianevski , A. K. Giri , and T. Aittokallio , “SynergyFinder 2.0: Visual Analytics of Multi‐drug Combination Synergies,” Nucleic Acids Research 48 (2020): W488–W493. 32246720 10.1093/nar/gkaa216 PMC7319457

[21] P. Koppula , L. Zhuang , and B. Gan , “Cystine Transporter SLC7A11/xCT in Cancer: Ferroptosis, Nutrient Dependency, and Cancer Therapy,” Protein & Cell 12 (2021): 599–620. 33000412 10.1007/s13238-020-00789-5 PMC8310547

[22] X. Liu , Z. Huang , Q. Chen , et al., “Hypoxia‐induced Epigenetic Regulation of miR‐485‐3p Promotes Stemness and Chemoresistance in Pancreatic Ductal Adenocarcinoma via SLC7A11‐mediated Ferroptosis,” Cell Death Discovery 10 (2024): 262. 38811540 10.1038/s41420-024-02035-x PMC11137092

[23] C. Springfeld , D. Jäger , M. W. Büchler , et al., “Chemotherapy for Pancreatic Cancer,” La Presse Médicale 48, no. 3 (2019): e159–e174, 10.1016/j.lpm.2019.02.025. 30879894

[24] W. Huanwen , L. Zhiyong , S. Xiaohua , R. Xinyu , W. Kai , and L. Tonghua , “Intrinsic Chemoresistance to Gemcitabine Is Associated with Constitutive and Laminin‐induced Phosphorylation of FAK in Pancreatic Cancer Cell Lines,” Molecular Cancer 8 (2009): 125. 20021699 10.1186/1476-4598-8-125 PMC2806309

[25] M. Roy‐Luzarraga and K. Hodivala‐Dilke , “Molecular Pathways: Endothelial Cell FAK—A Target for Cancer Treatment,” Clinical Cancer Research 22 (2016): 3718–3724. 27262114 10.1158/1078-0432.CCR-14-2021 PMC5386133

[26] F. Jiang , K. Jia , Y. Chen , et al., “ANO1‐mediated Inhibition of Cancer Ferroptosis Confers Immunotherapeutic Resistance through Recruiting Cancer‐Associated Fibroblasts,” Advanced Science 10 (2023): 2300881, 10.1002/advs.202300881. 37341301 PMC10460848

[27] F. He , P. Zhang , J. Liu , et al., “ATF4 suppresses Hepatocarcinogenesis by Inducing SLC7A11 (xCT) to Block Stress‐related Ferroptosis,” Journal of Hepatology 79 (2023): 362–377. 36996941 10.1016/j.jhep.2023.03.016 PMC11332364

[28] M. A. Badgley , D. M. Kremer , H. C. Maurer , et al., “Cysteine Depletion Induces Pancreatic Tumor Ferroptosis in Mice,” Science 368 (2020): 85–89. 32241947 10.1126/science.aaw9872 PMC7681911

[29] H. Mohammadi and E. Sahai , “Mechanisms and Impact of Altered Tumour Mechanics,” Nature Cell Biology 20 (2018): 766–774. 29950570 10.1038/s41556-018-0131-2

[30] K. J. Murphy , D. A. Reed , C. Vennin , et al., “Intravital Imaging Technology Guides FAK‐mediated Priming in Pancreatic Cancer Precision Medicine According to Merlin Status,” Science Advances 7, no. 40 (2021): abh0363. 10.1126/sciadv.abh0363 PMC8480933 34586840

[31] A. Osipov , A. B. Blair , J. Liberto , et al., “Inhibition of Focal Adhesion Kinase Enhances Antitumor Response of Radiation Therapy in Pancreatic Cancer through CD8^+^ T Cells,” Cancer Biology and Medicine 18 (2021): 206–214. 33628595 10.20892/j.issn.2095-3941.2020.0273 PMC7877172

[32] Q. Ding , W. Tang , X. Li , et al., “Mitochondrial‐targeted Brequinar Liposome Boosted Mitochondrial‐related Ferroptosis for Promoting Checkpoint Blockade Immunotherapy in Bladder Cancer,” Journal of Controlled Release 363 (2023): 221–234. 37717657 10.1016/j.jconrel.2023.09.024

[33] W. Wang , M. Green , J. E. Choi , et al., “CD8^+^ T Cells Regulate Tumour Ferroptosis during Cancer Immunotherapy,” Nature 569 (2019): 270–274. 31043744 10.1038/s41586-019-1170-y PMC6533917

[34] P. Liao , W. Wang , W. Wang , et al., “CD8^+^ T Cells and Fatty Acids Orchestrate Tumor Ferroptosis and Immunity via ACSL4,” Cancer Cell 40 (2022): 365–378. 35216678 10.1016/j.ccell.2022.02.003 PMC9007863

[35] M. Qiao , F. Zhou , X. Liu , et al., “Targeting Focal Adhesion Kinase Boosts Immune Response in KRAS/LKB1 co‐mutated Lung Adenocarcinoma via Remodeling the Tumor Microenvironment,” Experimental Hematology & Oncology 13 (2024): 11. 38291516 10.1186/s40164-023-00471-6 PMC10826079

[36] H. Song , T. Lu , D. Han , et al., “YAP1 Inhibition Induces Phenotype Switching of Cancer‐Associated Fibroblasts to Tumor Suppressive in Prostate Cancer,” Cancer Research 84 (2024): 3728–3742. 39137404 10.1158/0008-5472.CAN-24-0932 PMC11565174

