# Supplementary material for: Inhibition of Focal Adhesion Restricts Chemoresistance in Pancreatic Cancer by Targeting SLC7A11 Mediated Ferroptosis
Source: Adv Sci (Weinh). 2026 Apr 20:e75216. Online ahead of print. doi: 10.1002/advs.75216 (PMC13334639; doi:10.1002/advs.75216)
Supplement: Supplementary file 1 — Supporting File: advs75216‐sup‐0001‐SuppMat.docx. [file ADVS-9999-e75216-s001.docx]

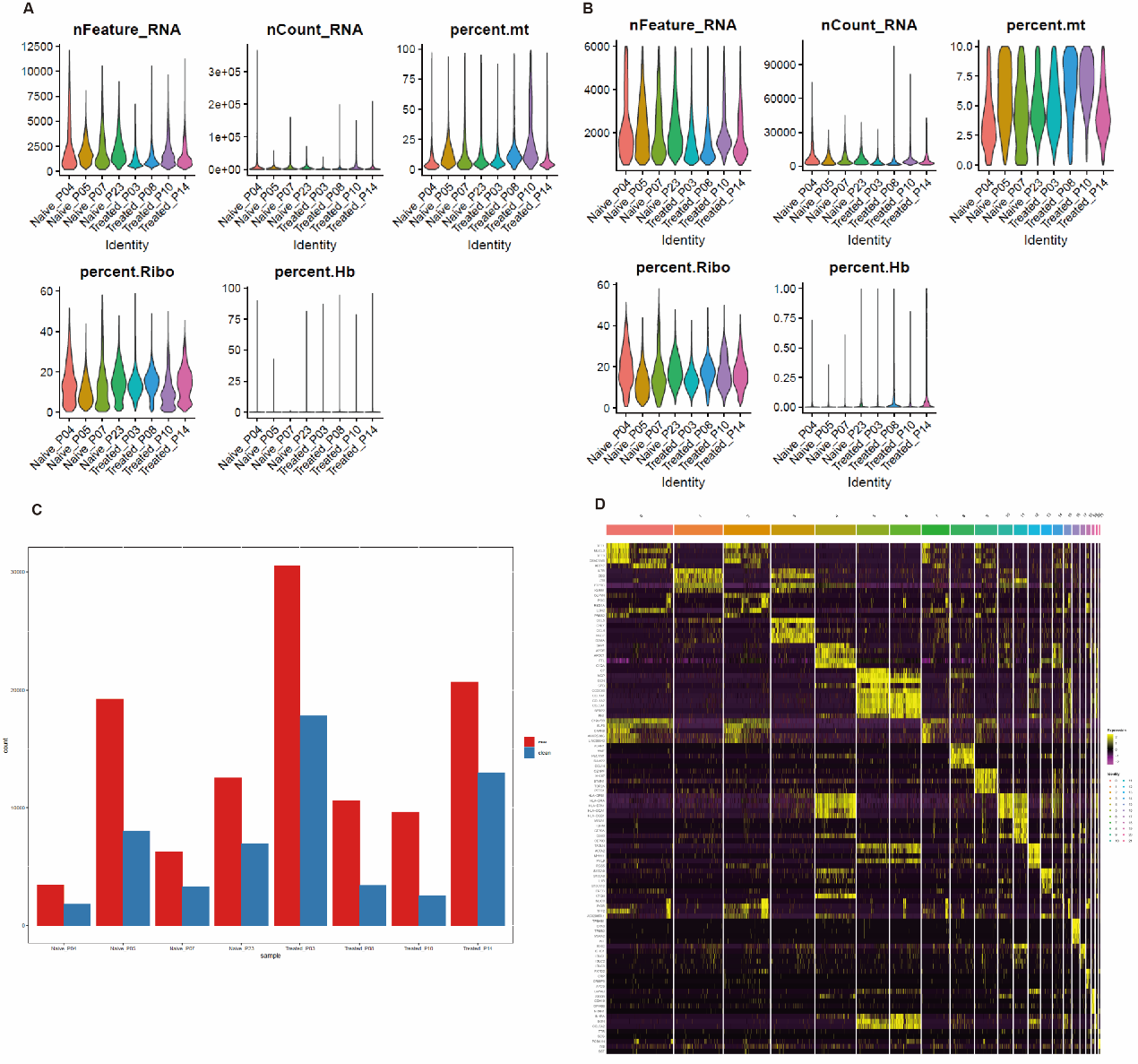


**Supplementary Figures S1**. (A-C) Pre- and post-quality control records in the scRNA-seq analysis. (D) Heatmap of all clusters and the top-10 marker genes.


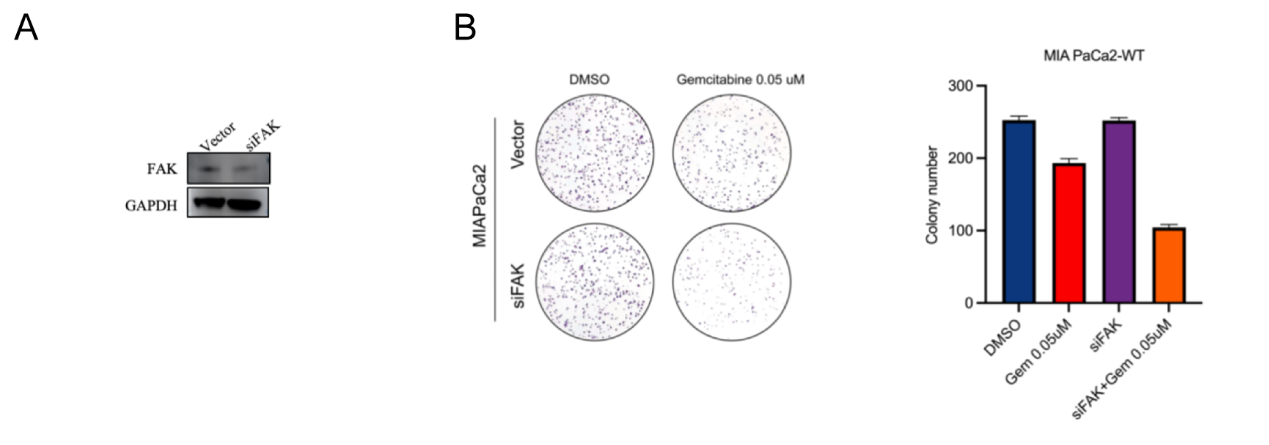


**Supplementary** **Figures S2.** Knockdown of FAK enhanced the sensitivity of gemcitabine for pancreatic cancer. (A) Knockdown efficiency verification of FAK; (B) Colony forming assays showed that knockdown of FAK enhanced the sensitivity of gemcitabine.


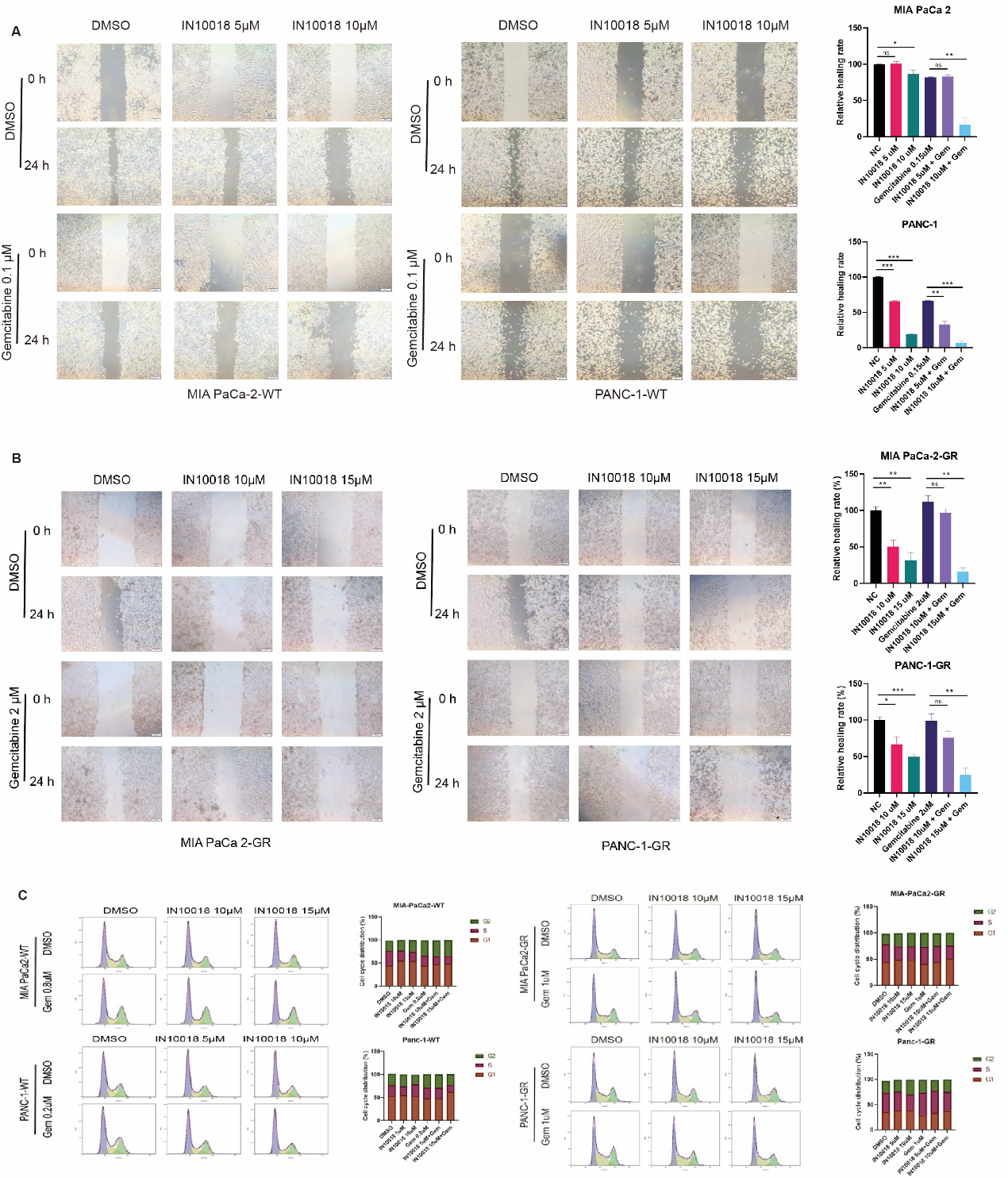


**Supplementary Figures S3.** (A-B) Wound healing assays in WT and GR cell lines treated with DMSO, IN10018 (5 and 10 μM for WT, 10 and 15 μM for GR), Gem (0.1 μM for WT, 2 μM for GR), and combination therapy. (C) Flow cytometry analysis of cell cycle distribution and the percentage of cells in G1, S, and G2 phase of each cell line.


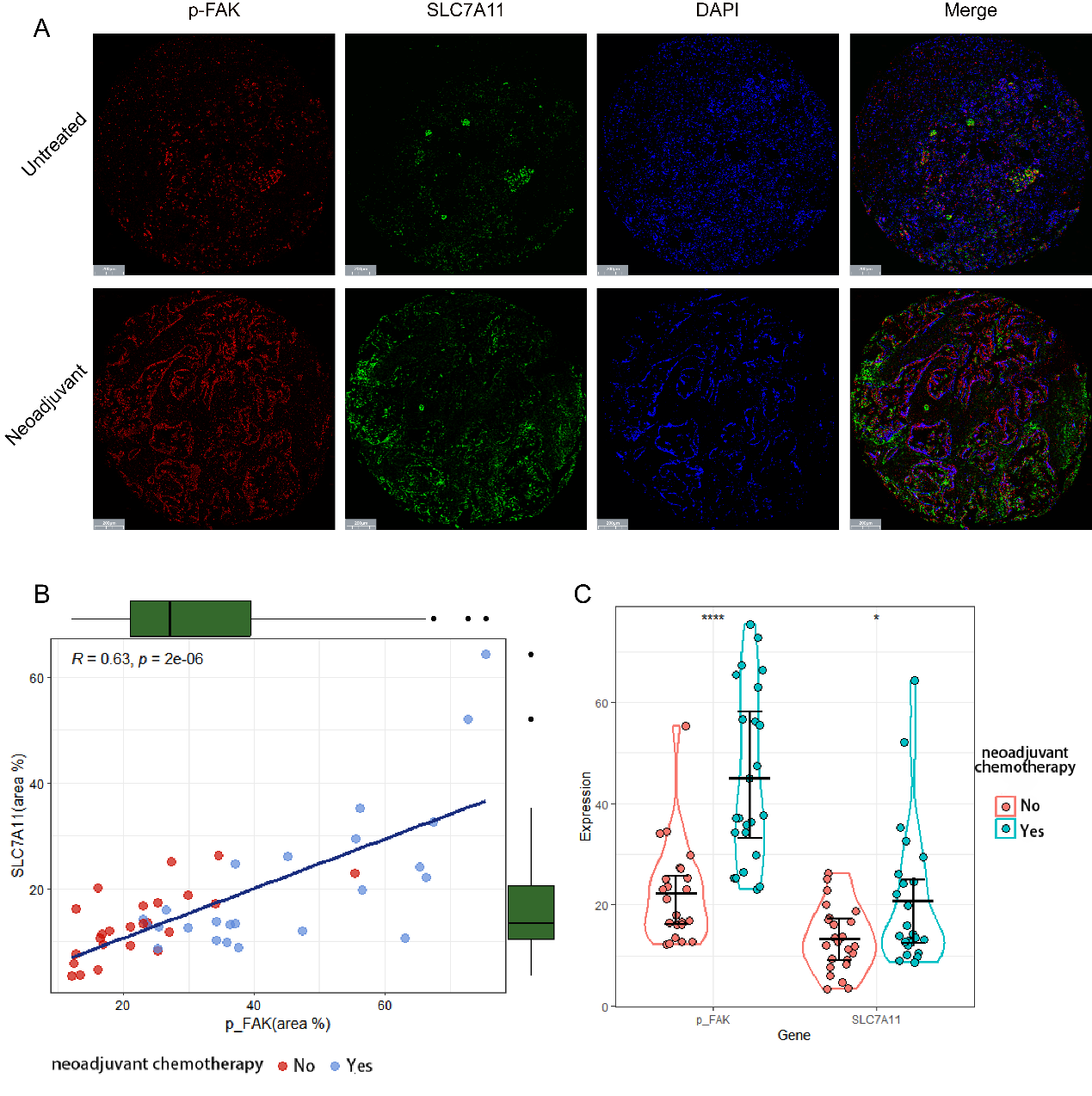


**Supplementary Figures S4**. Dual immunofluorescence analysis of p-FAK and SLC7A11 expression and their correlation in pancreatic cancer tissue microarrays from patients with or without neoadjuvant chemotherapy. **(A)** Representative immunofluorescence images of p-FAK and SLC7A11 staining. **(B)** Correlation analysis between p-FAK and SLC7A11 expression levels; blue and red dots indicate samples from chemotherapy-treated and untreated patients, respectively. **(C)** Comparison of p-FAK and SLC7A11 expression levels between chemotherapy and non-chemotherapy groups.


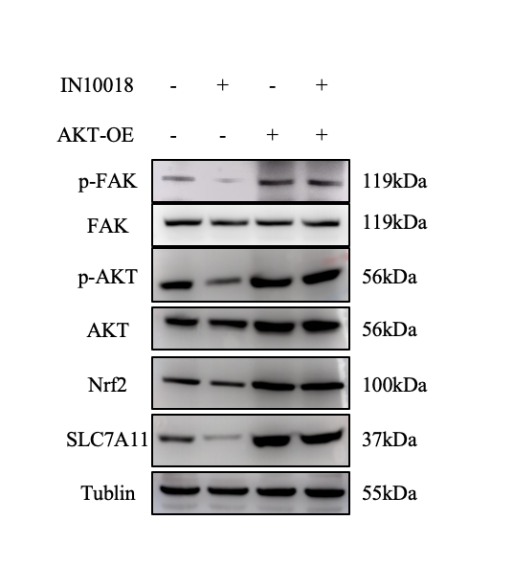


**Supplementary Figures S5.** Western blotting showed that overexpression of AKT could rescue the down regulation of SLC7A11 by IN10018, which illustrated the molecular mechanism of IN10018 regulating SLC7A11 through the PI3K-Akt signaling pathway.

**Supplementary Table S1**

**Sequences of lentivirus or plasmids used in this study.**

| shRNA plasmid | | primers |
| --- | --- | --- |
| SLC7A11-sh1-F | CCGGGCAGCTACTGCTGTGATATCCCTCGAGGGATATCACAGCAGTAGCTGCTTTTTG | |
| SLC7A11-sh1-R | AATTCAAAAAGCAGCTACTGCTGTGATATCCCTCGAGGGATATCACAGCAGTAGCTGC | |
| SLC7A11-sh2-F | CCGGGCACCCTTTGACAATGATAATCTCGAGATTATCATTGTCAAAGGGTGCTTTTTG | |
| SLC7A11-sh2-R | AATTCAAAAAGCACCCTTTGACAATGATAATCTCGAGATTATCATTGTCAAAGGGTGC | |

| Overexpression plasmid primers | |
| --- | --- |
| SLC7A11-ov-F | CGCAAATGGGCGGTAGGCGTG |
| SLC7A11-ov-R | CTGATTATGATCTAGAGTCG |

**Products used for immune cell populations and phenotypes analyses.**

| Product Name | Product ID | Company |
| --- | --- | --- |
| Brilliant Violet 510 anti-mouse CD3e | 100353 | BioLegend |
| mCD4-R711-FITC | 50134-R711-F | SB |
| Brilliant Violet 785™ Anti-Mouse CD8a | 100750 | BioLegend |
| APC Anti-Mouse CD45 | 147708 | BioLegend |
| PE-CF594 Rat Anti-mouse CD11b | 562287 | BD |
| APC-CY7 Rat Anti-Mouse Ly-6C | 560596 | BD |
| BV421 Rat Anti-Mouse Ly-6G | 562737 | BD |
| BV605 anti-Mouse F4/80 (lsotype: Rat lgG2a,k) | 123133 | BioLegend |
| Brilliant Stain Buffer Plus | 566385 | BD |
| Pharm Lyse | 555899 | BD |
| Anti-Rat and Anti-Hamster lg/Negative Control | 552845 | BD |
